# Matrix-entrapped cellular secretome rescues diabetes-induced EPC dysfunction and accelerates wound healing in diabetic mice

**DOI:** 10.1371/journal.pone.0202510

**Published:** 2018-08-28

**Authors:** Rucha Deshpande, Meghana Kanitkar, Sheetal Kadam, Kadambari Dixit, Hemlata Chhabra, Jayesh Bellare, Savita Datar, Vaijayanti P. Kale

**Affiliations:** 1 National Centre for Cell Science, NCCS Complex, University of Pune Campus, Ganeshkhind, Pune, Maharashtra, India; 2 Prof. Ramkrishna More Arts, Commerce and Science College, Akurdi, Pune, Maharashtra India; 3 Department of Chemical Engineering, Indian Institute of Technology-Bombay, Powai, Mumbai, Maharashtra, India; 4 Department of Zoology, S.P.College, Pune, Maharashtra India; Centro Cardiologico Monzino, ITALY

## Abstract

Cellular secretory products have infinite potential, which is only recently explored for research and therapeutic applications. The present study elaborated on the formation of a unique matrix-entrapped cellular secretome (MCS), a hydrogel-like secretome produced by bone marrow-derived mononuclear cells when cultured on a three-dimensional electrospun nanofiber matrix under specific conditions. These culture conditions support the growth of a mixed population predominantly comprising of endothelial precursor cells (EPCs), along with mesenchymal stromal cells and pericytes. Interestingly, such secretome is not formed in a pure culture of EPCs on the similarly formulated matrix, suggesting that a heterotypic cell-cell interaction is essential for the formation of MCS. In addition, the specific composition of the matrix was found to be a critical necessity for the formation of MCS. Furthermore, the application of the MCS as a substrate promotes the growth of EPCs in culture. It also rescues the diabetes-induced EPC dysfunction as assessed based on the parameters, such as viability, proliferation, colony formation, cellular adhesion, chemotactic migration, and tubule formation. MCS augments the levels of eNOS-specific mRNA (*Nos3*) and also promotes the restoration of the SDF1/CXCR4 axis in diabetic EPCs. Notably, a topical application of MCS on diabetic wounds leads to an accelerated wound closure. Thus, the current data showed that MCS forms an excellent cell-free biomaterial in the treatment of diabetic wounds and non-healing ulcers.

## Introduction

Biomaterials have revolutionized the field of drug delivery and applications and are categorized as scaffolds, meshes, matrices, hydrogels and substrates. Some of the routinely used scaffolds are collagen-derived matrices [1], silk-based meshes/matrices [2, 3], dextran hydrogels [4], and electrospun nanofiber matrices such as poly-L-lactic acid (PLLA) [5, 6]; of which, electrospun nanofiber matrices are preferred in biological applications. These scaffolds provide a three-dimensional (3D) architecture, which mimics the extracellular matrix (ECM)-like nano-architecture, mechanical strength, and a surface suitable for various cellular interactions [7,8]. These matrices are potential candidates for skin implantation and wound healing as they show tensile strength similar to that of the skin. The resemblance to ECM-like structure allows enhanced cellular growth and provides a niche for cell-cell crosstalk [9, 10].

Recent studies have shown that the cells and their secretomes play a critical role in the regenerative processes. Although derived from ‘biological sources,’ secretomes are not perceived as ‘classic utilizable biomaterials’. Conversely, they are used for assessing the ability to control and regulate the natural biological processes. They are also used in the detection and diagnosis of diseases, such as myocardial infarction and cancer [11–13].

Cellular secretomes, containing trophic cytokines, immunomodulatory molecules, and proteins, are typically collected as conditioned media (CM) from various short- and long-term cell cultures[14], thereby limiting their scope of delivery and direct applicability. Thus, capturing these secretomes in a natural hydrogel form might resolve the delivery and application issues.

Mesenchymal stem cells (MSCs) exhibit the ability of regeneration through the secretomes [15]. Typically, the CM of MSCs is collected and concentrated for therapeutic applications. Therefore, the component integrity, efficacy, and activity are compromised during processing [16]. Such downstream processing steps not only affect the quality of the final product but also increase the production cost and the contamination risk.

Thus, devices that facilitate entrapment and harvesting of the secretory components without the loss of activity are needed for enhanced efficacy of harvesting. However, presently, such devices are not available. Hence, in the present study, we describe a simple, yet ingenious, culture system. It comprises of electrospun polycaprolactone-gelatin nanofiber matrix (PCG matrix) used in conjunction with a mixture of growth media for the culture of bone marrow-derived mononuclear cells (BM-MNCs). As a result, the system yields a mixed cell population primarily containing EPCs as the major cell population with a small, but significant percentage of MSCs and pericytes. Notably, the system allows entrapment of cellular secretome in the form of a hydrogel. The unique feature of this system is that this secretome can be harvested easily and applied directly to the wounds without any downstream processing.

We demonstrate here, for the first time, the formation of a novel MCS that can be used for direct application in research and clinics without any downstream processing. It can also be used as a “biomaterial with hydrogel-like consistency.” MCS presents the unique ability to support EPCs that might be attributed to its rescue effect on diabetic EPCs leading to accelerated wound healing. Taken together, the current data designated MCS as a major leap in topical applicatory products for wounds, lesions, and other vasodilatory defects.

## Materials and methods

### Drugs, chemical reagents, and materials

Endothelial cell growth medium (EGM-2 Single Quots) was purchased from Lonza (Walkersville, MD, USA), while fetal bovine serum (FBS) and Dulbecco’s Modified Eagle’s medium (DMEM)were purchased from Invitrogen Life Technologies (Carlsbad, CA, USA).3-(4,5-dimethylthiazol-2-yl)-2,5- diphenyltetrazolium bromide (MTT) and vitronectin (VN) from rat plasma werepurchased from Sigma–Aldrich, (St Louis, MO, USA). All plastic ware were purchased from BD Falcon (Bukit Batok Crescent, Singapore).

#### Fabrication of PCG nanofiber matrix

Polycaprolactone (PC) (Sigma–Aldrich) with an average molecular weight of 80,000 Da and gelatin (type A) from porcine skin were used together with1,1,1,3,3,3-hexafluoro-2-propanol (HFP) (Sigma–Aldrich). PC/gelatin in a 3:1 ratio (w/w) was solubilized in HFP to obtain a 12% solution and electrospun into nanofibers. For electrospinning, the polymer solution was filled in a 5 mL plastic syringe (BD, India) connected to a blunt-end metallic needle (24 Gauge). Then, the syringe was loaded into a syringe pump (New Era Pump System Inc., Farmingdale, New York, USA) and electrospun at the rate of 1 mL/h to obtain 400–700nm fibers. The collector was covered with an aluminum foil. The distance between the needle tip and the collector was maintained at 12.5 cm, and a voltage of14 kV was applied. The entire process was conducted in a fume hood at ambient temperature of 27°C and 55% relative humidity. The electrospun scaffold was maintained under vacuum overnight before the subsequent experiments. The matrix was sterilized with gamma radiation (8000rads). Fabricated PCG matrix consists of randomly oriented smooth nanofibers, 400–700nm in diameter [17].

#### Animals

All animal procedures used in the study complied with the international guidelines governing animal experimentation and were approved by the Institutional Animal Ethics Committee at NCCS, Pune, India (certificate number: EAF/2012/B-180).

Swiss/albino male/female mice (6–8-week-old, weighing at least 25 g), purchased from Jackson Laboratory (USA) were bred in an inbred colony at the National Centre for Cell Science (NCCS), India, for the experimental use. The animals housing facilities were maintained at 12h day/night cycles, 25°C ambient temperature, and 50% humidity and allowed free access to standard feed and water.

#### Induction of experimental diabetes, wound creation, and closure

Mice were rendered diabetic by an intraperitoneal injection of the β-cell-specific toxin, streptozotocin (STZ; 140 mg/kg; Sigma-Aldrich). STZ was reconstituted in chilled sodium citrate buffer (pH 4.5) just before the injection. 7–10 days post injection, random plasma glucose concentrations were estimated. Mice with plasma glucose concentrations >200 mg/dL for at least 2 weeks were considered to be distinctly diabetic.

For wound creation, the animals were de-haired and anesthetized before wounding to minimize the pain. Full-width wounds of 0.75 cm diameter were created on the flanks of diabetic and control mice. Subsequently, the wounds were washed with antibiotic (fluconazole, 2 mg/mL, Cipla, Roorkee, India), and MCS (10μg/mL) was applied topically every 48 h for 8days; this was termed as the MCS-treated diabetic group or the test group. The wounds were photographed every alternate day for obtaining the wound closure measurements under identical settings. The animals were monitored daily under the guidance of the veterinarian and observed for signs of infection, food and water consumption, bedding status, and comfort. The experimental animals did not display any adverse clinical symptoms following wound creation. At the end of the experiment, the animals were euthanized by CO_2_ asphyxiation. Data were represented as % wound closure. A minimum of 5–6 mice was utilized per group, and the experiment repeated three times.

#### Study of cellular profile during wound healing

For determination of the cellular profile and the quality of wound healing, non-diabetic (ND), diabetic (D), and MCS-treated wounds were collected on days 2, 4, 6, 8, and 10post-wounding. Mice were sacrificed by CO_2_ asphyxiation just before harvesting the wound biopsies. The paraffin-embedded wound sections were subjected to hematoxylin-eosin (HE) staining and analyzed. Images were representative of up to 4 mice per group.

#### Harvesting of MCS

Mice were sacrificed by CO_2_ asphyxiation and femurs extracted aseptically. These bones were flushed using plain DMEM; the cellular suspension was loaded onto HiSep LSM (HiMedia, Mumbai, India) and centrifuged for 30 min at 1500 rpm and room temperature. Buffy coat was aspirated and washed two times with plain DMEM for 15 min at 1500 rpm and room temperature. Then, these cells were enumerated, and complete mononuclear cell (MNC) fraction (viability >98% as determined by trypan blue dye exclusion test) was seeded onto PCG matrix and cultured in a medium comprising 1:1 DMEM + 10% mesenFBS + EGM-2 + 20% FBS at 37°C, 5% CO_2_ for 14 days. The medium was replenished every 48 h. After 14 days of culture, CM was collected separately and the entrapped cell secretome harvested mechanically using sterile glass rolling mechanical extractor by gently pressing the matrix. The collected secretome and CM were stored at -80°C.

This PCG formulation and culture process for MCS formation are under IPR (IPA no.1951/MUM/2014).

#### Characterization of PCG post-MCS formation using scanning electron microscopy (SEM)

After 14 days of culture, the cells along with the matrix were fixed with 2.5% glutaraldehyde. On the day of SEM analysis, matrix harboring the fixed cells was washed with PBS, dehydrated with graded ethanol from 10–100%, coated with platinum and observed under SEM (JSM 7600F, JEOL, Japan).

#### Structural characterization of MCS by cryo-SEM

A portion of MCS harvested from the matrix was freeze-fractured, coated with platinum, and observed under cryo-SEM (JSM 7600F, JEOL, Japan).

#### Identification of cell population involved in MCS formation

After 14 days of culture, the cells along with the PCG-matrix were washed with 1X PBS and incubated with Alexa flour 488-labeled Dil-Ac-LDL, an EPC-specific marker, for 3 h at 37°C, washed, and fixed with chilled 2% buffered paraformaldehyde (PFA). Then, the matrix was stained with antibodies to CD90 (for MSCs) or CD146 (VCAM-1), and CD31 and Ang-1 (for pericytes). The secondary staining was effectuated with anti-mouse Cy3, anti-goat Cy5, and anti-rabbit FITC. DAPI was used for nuclear staining. All dual positive cells—EPCs/MSCs/pericytes and unstained cells—were counted manually in at least 10 randomly selected non-overlapping fields per sample, using an inverted fluorescence microscope and Image-pro software (Leica).Data were denoted as a percentage of cells of each cell type ± SD per field per sample.

#### Isolation, culture, and characterization of non-diabetic and diabetic mouse EPCs

The EPC isolation protocol was followed as described previously with minor modifications [18]. Briefly, 1×10^6^ BM-MNCs were cultured in DMEM + 10% FBS at 37°C with 5% CO_2_ for 48h. Subsequently, the non-adherent cells were removed and seeded onto either VN-coated wells (0.5 μg/mL; VN-EPCs: control group) or MCS-coated wells (1 μg/mL; MCS-EPCs: experimental group). The cells were cultured in EGM2 +20% FBS for 14 days at 37°C, 5% CO_2_, and the media was replenished every 48 h.

#### Cell adhesion assay (CAA)

EPCs (1000 mMNCs/well; VN- or MCS-coated) were cultured in EGM-2 supplemented with 20% FBS for 14 days at 37°C and5% CO_2_, followed by washing and staining with crystal violet. Subsequently, the cells were washed, the dye extracted with 5%SDS, and the absorbance of the solution measured at 595nm. At least three assays were performed for each sample. Data were expressed as mean absorbance/1000EPCs ± standard deviation (SD).

#### Colony formation assay (CFU)

EPCs (1000 mMNCs /well, VN- or MCS-coated) were cultured as described above. After day 14, the number of colonies was counted manually using a phase contrast microscope (Olympus, Center Valley, PA, USA). A group of ≥10 cells was considered as a colony. The assay was performed at least three times (N = 3) for each sample. Data were expressed as mean CFU/1000EPCs ±SD.

#### Proliferation assay

Proliferation of EPCs cultured on either VN (VN-EPCs) or MCS (MCS-EPCs) was determined by 3-(4, 5-dimethylthiazol-2-yl)-2,5- diphenyltetrazolium bromide (MTT) assay. Day 14 EPC cultures were incubated with MTT (0.5 mg/mL, Sigma) for 4 h in the dark at 37°C and 5% CO_2_. The blue-purple formazan crystals were solubilized in acidified isopropanol and absorbance measured at 550/650 nm. The assays were performed where N was at least 4. Data were expressed as mean % proliferation ± SD.

#### Viability assay

The viability of VN- and MCS-EPCs was assessed using trypan blue dye exclusion test. Data were expressed as % viability ± SD.

#### Migration assay

The migratory potential of EPCs was evaluated by a modified Boyden chamber (BD) assay. Briefly, day 14 VN- and MCS-EPCs were detached from wells using trypsin phosphate versene glucose (TPVG) (HiMedia), counted, and placed in the upper chamber of 24-well Transwell plates (8μm pores) (500 EPCs/well) in serum-free DMEM. Serum-free DMEM containing vascular endothelial growth factor (VEGF) (50ng/mL) was placed in the lower chamber. After 24 h incubation, the upper surface of the membranes was gently cleaned with a cotton bud. Subsequently, the membranes were washed with 1X PBS, fixed with chilled 2% PFA, and stained with crystal violet for 3h. The number of migrated cells was counted manually under an inverted light microscope (Olympus) and data expressed as % EPC migration ± SD.

#### Tubule formation assay

EPCs were cultured on VN or MCS for 14 days in EGM-2 + 20% FBS. On day 14, the medium was changed to DMEM +10% FBS and the culture continued for an additional 72h. Subsequently, the number of cells with or without tubules was scored independently in at least 10 non-overlapping fields. Data were expressed as mean % tubule formation ± SD.

### Evaluation of gene expression

Total RNA was extracted from EPCs or wound biopsies using TRIzol method (Invitrogen Life Technologies). Briefly, EPCs or wound biopsies were incubated with 750μLTRIzol reagent overnight at 4°C. Then, 200 μL chloroform was added to the cell/tissue lysate, and the suspension centrifuged at 10,000rpm for 16min at 4°C. The upper aqueous layer was collected, mixed with 350 μL isopropanol, and incubated at the ambient temperature for 15 min, followed by centrifugation at 10,000rpm for 15min at 4°C. The resulting pellet was washed with 70% ethanol, air dried, and solubilized in nuclease-free water. The concentration of RNA was measured using Nano-drop (ND-1000, UV/Vis spectrophotometer, Nanodrop Technologies, Wilmington, DE, USA). Subsequently, cDNA was prepared from 200ng total RNA using Applied Biosystems kit according to the manufacturer’s instructions. The PCR conditions for 30 cycles of amplification were as follows: 95°C (3 min), annealing temperature specific for each set of primer (45 s), 72°C (1 min) (EP Cycler 5, Eppendorf India Limited, Ambattur, Chennai, India) along with the final extension of 5 min at 72°C. The PCR products were resolved on 1.2% agarose gels and visualized using ethidium bromide (EtBr). The primer sequences were as follows: *β-actin* (Forward:5’-GGAATCCTGTGGCATCCA-3’, Reverse: 5’-TAACAGTCCGCCTAGAAGCA-3’);*Sdf1a*(Forward: 5’-GCTCTGCATCAGTGACGGTA-3’,Reverse: 5’-ATCTGAAGGGCAACGTTTGG-3’);*Cxcr4* (Forward: 5’-TCAGTGGCTGACCTCCTCTT-3’, Reverse: 5’-CTTGGCCTTTGACTGTTGGT-3’); *Vegfr2*(Forward: 5’-CCAGAGATTCCATGCCACTT-3’, Reverse: 5’-GTGACCAACATGGAGTCGTG-3’); *TNF-α* (Forward: 5’-GCGAGGTGGAACTGGCAGAAG-3’, Reverse: 5’-GGTACAACCCATCGGCTGGCA-3’); *IL-1β* (Forward: 5’-TCATGGGATGATGATGATAACCTGCT-3’, Reverse: 5’ CCCATACTTTAGGAAGACACGGATT3’); *IFN-γ* (Forward: 5’-GAGGAAAACAAGAGGACCC-3’), Reverse: 5’-GGAACCAGATTTTGATGTGTC-3’); KL (Forward: 5’-GCATCTCTACAACACCTCTT-3’, Reverse: 5’-GCAGAAGAGACGAGAGGT-3’). The bands were observed using the gel documentation system (Syngene G:Box, SanDiego, CA, USA), and the band intensities analyzed using the ImageJ software. The gene expression values were normalized against that of *β-actin* (housekeeping gene control).The assay was conducted on at least three samples collected from three independent experiments, and data plotted as mean ± SD.

#### Determination of nitric oxide (NO) levels

The level of NO was measured using Greiss reagent (0.1% Naphthylethylenediaminedihydrochloride + 1% sulfanilamide). CM (100 μL) from VN-/MCS-cultured ND (VN/MCS-ND-EPCs) and D-EPCs (VN/MCS-D-EPCs) was incubated with an equivalent volume of freshly prepared Greiss reagent and incubated in the dark for 30 min. Optical density was measured using spectrophotometer at 550 nm. The data were expressed as μM nitrites/1000EPCs. NaNO_2_was used as a standard.

#### Estimation of reactive oxygen species (ROS)

EPCs from each group were incubated with 10 μM DCFH-DA at 37ºC for 10 min in the dark, and the resulting fluorescence was measured using Fluroscan Ascent FL fluorimeter (Thermo Electron Corporation, Waltham, Massachusetts, USA). The excitation was at 438 nm and emission at 510 nm. All values were corrected by subtracting auto-fluorescence for respective groups.

#### MnSOD activity assay

CM of VN/MCS-ND and D-EPCs were collected on day14 and stored at -80°C until further use. The manganese superoxide dismutase (MnSOD) activity was measured using the SOD activity kit (Enzo Life Sciences, Farmingdale, NY, USA). Cu/Zn SOD enzyme activity from all cell lysates was inhibited with 2mM KCN. All procedures and data analyses were carried out according to the manufacturer’s instructions. Data were presented as units of MnSOD/mg of protein ± SD.

#### In-solution digestion and LC-MALDI TOF/TOF analysis

To identify the components present in MCS, in-solution digestion with trypsin was carried out, followed by mass spectroscopic analysis using LC-MALDI TOF/TOF analysis (Horth et al., 2006; Pendharkar et al., 2016), respectively [19, 20]. Briefly, MS spectra were acquired on a 4800 plus MALDI-TOF/TOF (AB Sciex, Foster City, USA)mass spectrometer equipped with a 200 Hz repetition rate Nd:YAGlaser in reflector positive ion mode in the mass range of 800–4000 m/z. A total of 900 laser shots for MS and 2500 for MS/MS were used to acquire the spectra. The data from the MALDI-TOF/TOF was analysed by the GPS™ Explorer version 3.6 (AB Sciex) using MASCOT search engine in mouse taxonomy by applying following parameters: MS tolerance: 75 ppm; MS/MS tolerance: 0.4 Da; enzyme: trypsin; fixed modification: carbamidomethyl; variable modifications: oxidation (methionine) and deamidation (N, Q).Proteins were identified using Swiss Prot database.

#### Data analysis and statistics

Data were expressed as mean ± standard deviation (SD). Statistical comparisons between the groups were performed using one-way ANOVA. P<0.001 was considered to be significant as determined by the Tukey post-hoc analysis.

## Results

### PCG-matrix provides 3D architecture essential for secretome entrapment

**Fig 1:**
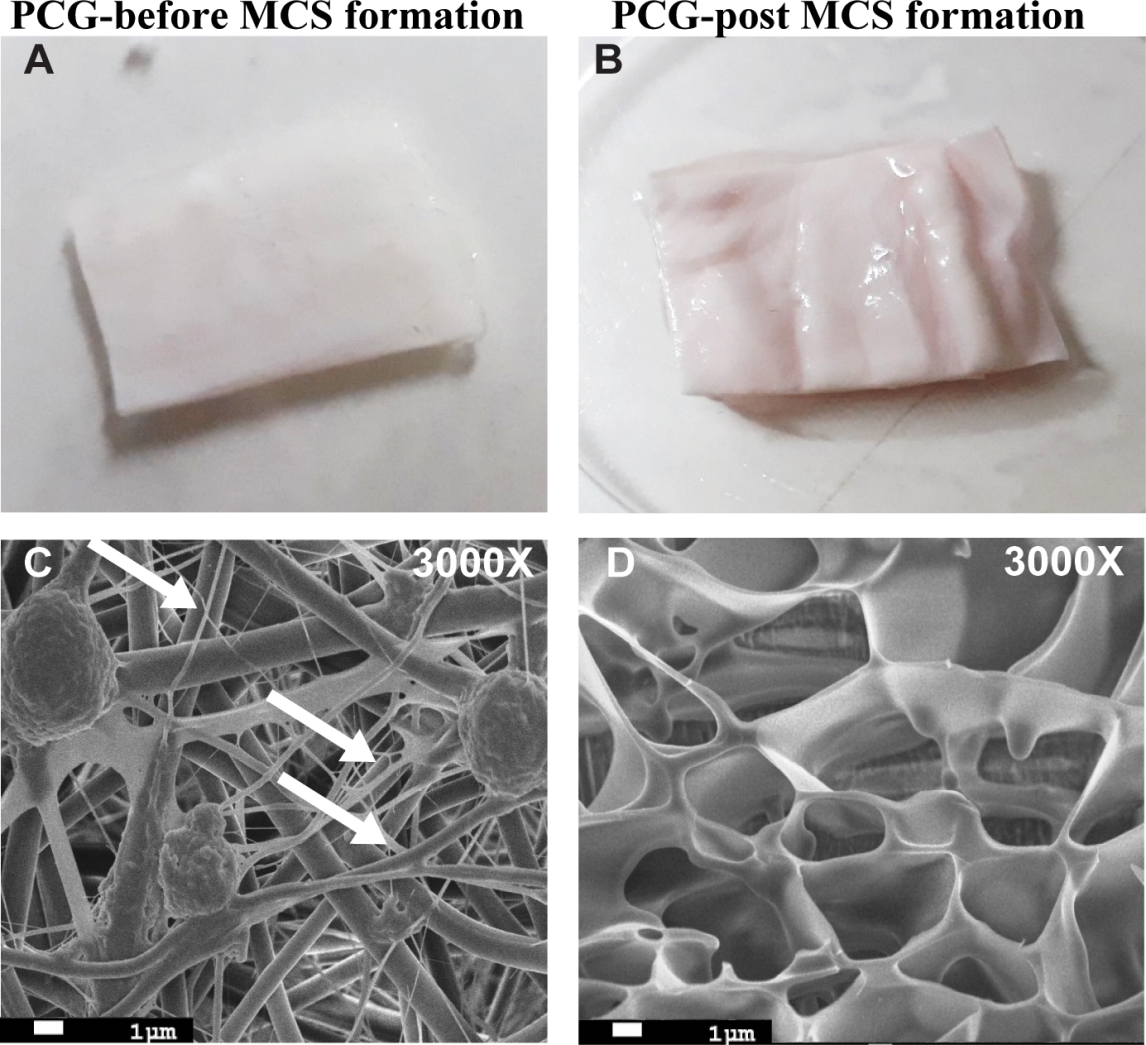
Secretome formation and structural analysis. **(A, B)** PCG-matrix before and after MCS formation, respectively. Note the turgidity of the matrix post MCS formation. **(C)** SEM image of the matrix post-MCS formation is illustrated. Cells cultured on PCG show intense cellular interactions as indicated by the white arrows. Magnification 3000X. Scale bar = 1μm. **(D)**. Cryo-SEM image shows ultra-structure of the MCS. Magnification 3000×. Scale bar = 1μm.

Previously we have demonstrated that 3D mesh like structure of PCG allows growth and adhesion of EPCs [17]. Such architecture may allow cell-cell crosstalk and may promote the formation of the hydrogel-like cell secretome. BM-MNCs cultured on PCG produced hydrogel like cell secretome: MCS. The produced hydrogel remains entrapped in the 3D meshwork of the matrix and causes the matrix to become turgid (Fig 1A and 1B).

PCG-matrix harboring MCS was fixed with 2% glutaraldehyde and observed by SEM. The microscopic images exhibited that the cells were firmly adhered to the scaffold and produced extensions (Fig 1C), confirming that the PCG-nanofiber matrix provides the necessary 3D architecture required for the mechanical support to the cells for attachment, proliferation, and cell-cell interaction (Fig 1C).

Furthermore identifying the source(s) of MCS is imperative as the starting cellular population was heterogeneous consisting of total BM-MNCs. Confocal microscopy analysis of entrapped cells revealed two principal populations: EPCs (62.75±3.4%) and MSCs (23.33±2.1%) with a small percentage of pericytes (11.11±4.9%) (S1A Fig). Only a minor population (2.81±0.8%) remained unidentified. The two major cell populations, EPCs and MSCs, might be the primary contributors to the formation of MCS. Control cultures comprising of homogenous cell populations of EPCs or MSCs alone did not lead to the formation of MCS under the present culture conditions (data not shown), indicating that the heterogeneity of the cell population was essential for the formation of MCS. Similarly, EPC-MSC co-cultures established without PCG matrix or established on other matrices, such as PLLA, also did not produce MCS (data not shown), indicating that both PCG matrix and specific media composition are crucial components for the formation of MCS.

#### Harvesting and characterization of MCS

MCS solubilizes easily in aqueous media, thereby allowing the possibility of solution extraction; however, this would have diluted the secretome, and thus, defeated the purpose. Hence, we determined whether it was possible to mechanically harvest the hydrogel from the meshwork. Thus, we created a glass rolling mechanical extractor that was used to physically force the hydrogel out of the meshwork in a sterile environment. We found that MCS can be harvested easily without dilution by this procedure. The harvested hydrogel was stored at -80°Cuntil further use. The protein content of MCS was determined using Micro BCA Kit (Pierce, Rockford, IL, USA).

The structural properties of MCS were analyzed using Cryo-SEM, while the chemical composition was analyzed using LCMS (Supplementary data). On the other hand, the structure was elucidated by processed the MCS and observing under cryo-SEM after freeze fracture. The ultra-structure of MCS exhibited interconnected 3D microporous architecture mimicking the native ECM structure (Fig 1D).The 3 D architecture of MCS is critical as it may provide a 3D biomimetic niche for the growth of various cell types and enhance cell-cell interactions.

#### MCS supports the growth of EPCs

Next, we assessed the potential of MCS to act as a substrate for attachment, growth, and proliferation of EPCs. MCS-EPCs were assayed using standard cellular assays, such as cell adhesion assay (CAA), colony forming unit assay (CFU), % viability assay (trypan blue dye exclusion test,), and proliferation index assay (MTT) and compared to the most routinely used substrate, VN. [21].

**Fig 2:**
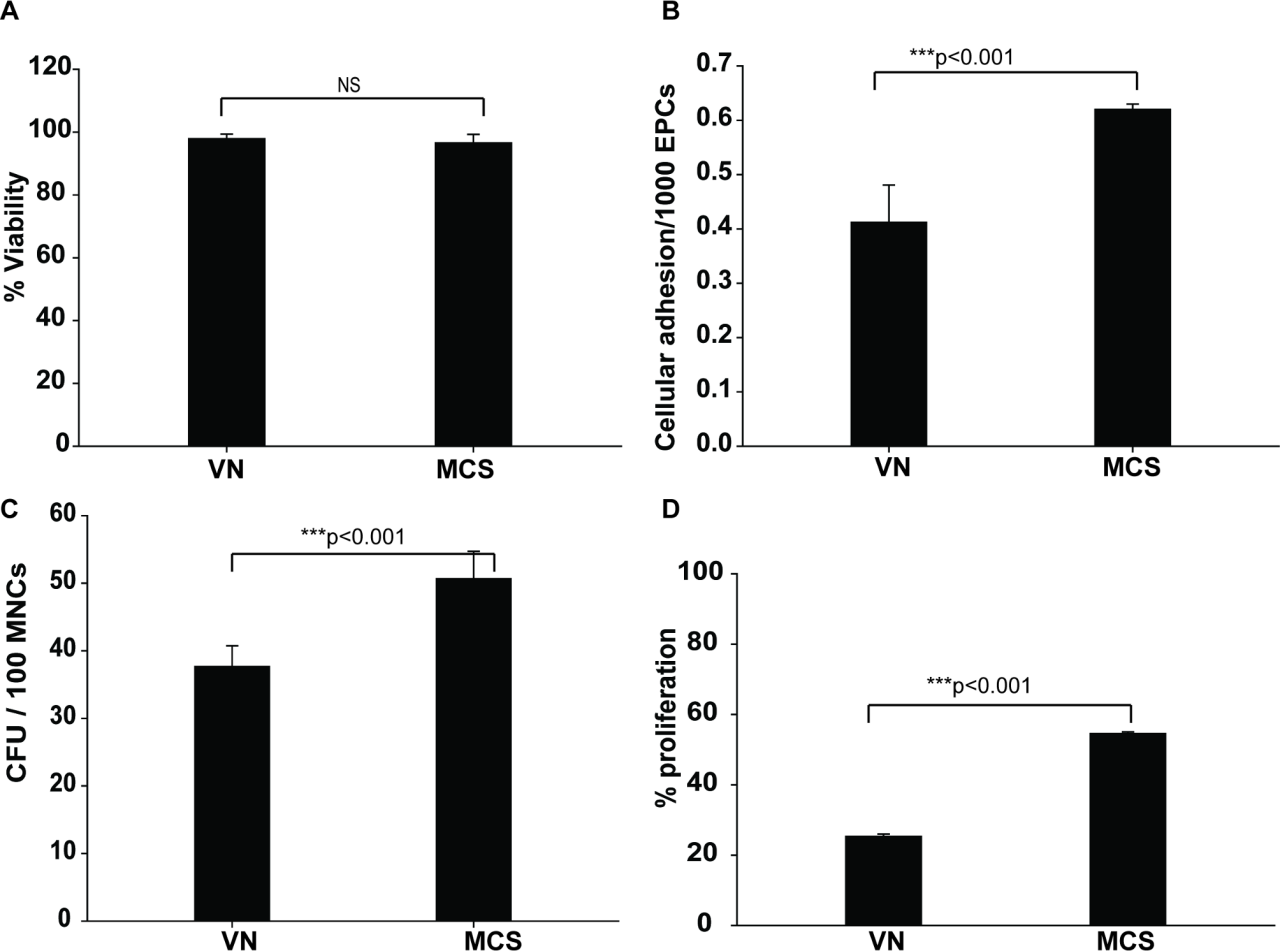
MCS is a superior substrate for EPC growth. Efficacy of MCS as a substrate for EPC growth was assessed by %viability **(A)**, cellular adhesion **(B)**, colony formation **(C),** and proliferation potential **(D)** of EPCs in comparison with VN. Data are represented as the mean of three independent experiments (N = 3) ± SD. ***P<0.001.

Although both VN and MCS support a prolonged (14 days) culture of isolated EPCs with equal efficiency in terms of % viability (Fig 2A), MCS was superior in terms of adhesion (1.5-fold higher; Fig 2B),colony formation (1.3-fold higher; Fig 2C), and proliferation potential(1.9-fold higher; Fig 2D) as compared to VN. These data highlighted the efficacy of MCS as a substrate for EPC growth. This phenomenon is especially vital as the EPCs are fastidious cells and small changes in media, pH, and growth substrate can markedly affect the overall cell yield. Moreover, this effect is pronounced with respect to EPCs obtained from diabetic sources. In The current study, the overall EPC yield from D mice is significantly lower than that derived from ND sources (ND: 855 ± 30/1000 MNCs; D: 677 ± 32/1000 MNCs; *P<0.001), when grown on standard VN-coated surfaces.

#### MCS augments D-EPC growth

Since MCS supported the superior growth of normal (ND) EPCs, we examined whether it would also support the growth, adhesion, and proliferation of EPCs isolated from diabetic mice (D-EPCs).

**Fig 3:**
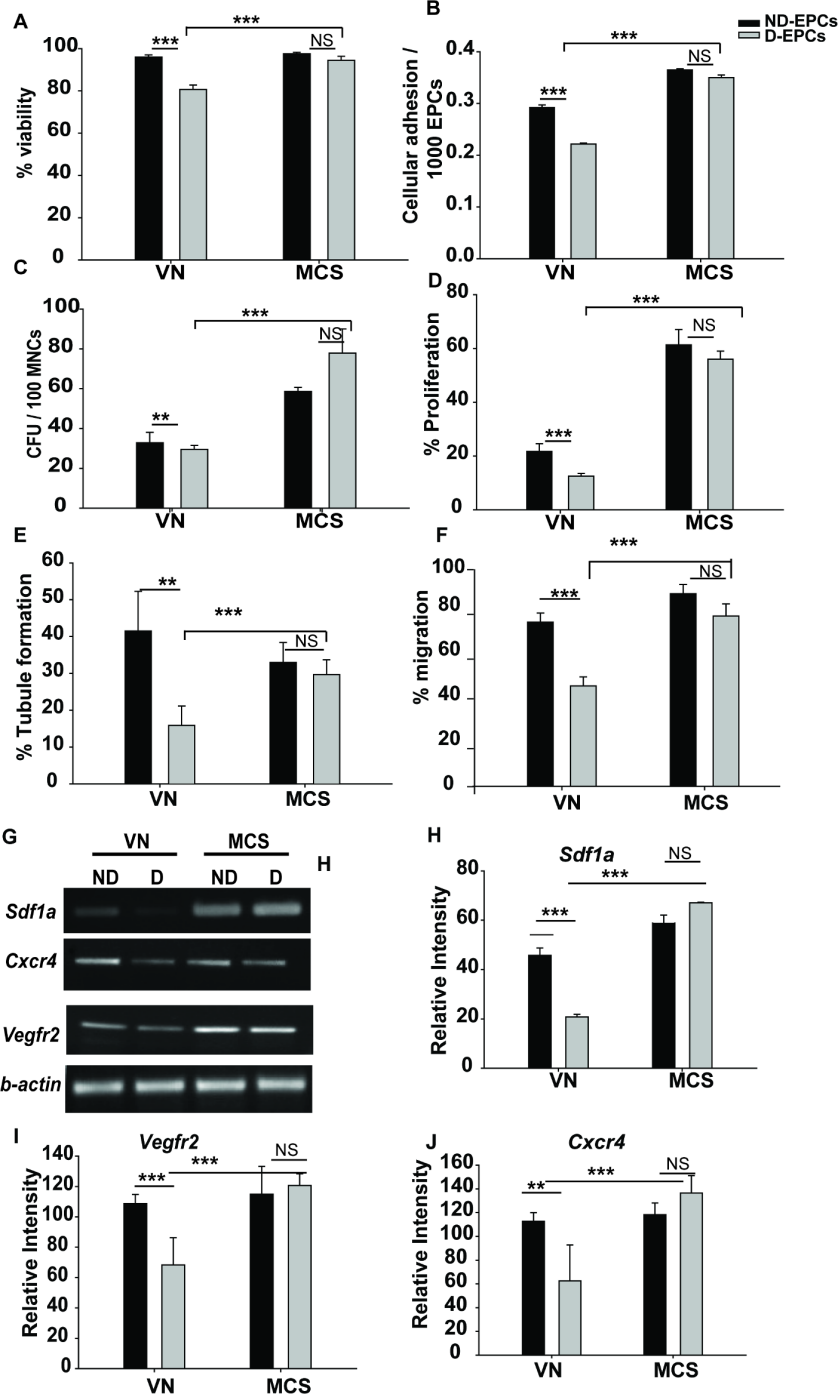
MCS augments D-EPC growth and restores their functionality. **(A-D)** Day14 D-EPCs cultured on MCS were assessed for % viability **(A),** cellular adhesion **(B)**, colony formation **(C),**and proliferation potential **(D)** in comparison with VN. The functionality of D-EPCs cultured on MCS was assessed by % tubule formation and % migration ability of D-EPCs **(E and F, respectively)**. MCS restores the functionality of D-EPCs by restoring the SDF-1α/CXCR-4/VEGFR-2 levels. (**G-J**)Reverse transcriptase- polymerase chain reaction (RT-PCR) analysis of *Sdf-1a*, *Cxcr-4*, and *Vegfr-2* is depicted. Densitometric analysis of the bands was conducted using ImageJ software and data were normalized with *β-actin*. Data are represented as mean ± SD (N = 3). **P<0.01, ***P<0.001, NS (not significant).

We found that VN did not support the optimal growth of D-EPCs, whereas MCS supported the growth of both ND- and D-EPCs with equal efficiency (Fig 3A). Moreover, MCS prevented the depletion of the normally deficient adhesion and colony formation ability of D-EPCs as compared to VN (Fig 3B and 3C, respectively). Surprisingly, the culture of D-EPCs on MCS was not beneficial in enhancing the proliferation potential of ND- as well as D-EPCs as compared to VN (Fig 3D). CM (liquid media around the matrix) collected during MCS formation was also tested for the ability to support the growth of ND-and D-EPCs. CM did not support ND- or D-EPC growth as observed from cellular assays (S1B–S1E Fig).

These data indicated that the active components are entrapped in the MCS and not released into the supernatant.

#### MCS promotes tubule formation and chemotactic migration ability of D-EPCs

Although the current data demonstrated a definite growth-related advantage of MCS for D-EPCs, it was still unclear if the functionality of these cells was improved. Hence, we performed tubule formation and chemotactic migration assays on the cultured VN- and MCS-ND/D-EPCs (indicators of EPC functionality) and found that VN-D-EPCs showed compromised tubule formation and % migration as compared to VN-ND-EPCs (Fig 3E and 3F; first two bars). This compromised tubule formation and migration potential were significantly rescued when D-EPCs were cultured on MCS, illustrating its role in the rescue of diabetes-induced EPC dysfunction (Fig 3E and 3F, respectively; grey bars).

These data demonstrated that MCS not only actively supports the growth of D-EPCs but also restores their functionality.

#### MCS restores SDF-1 α-CXCR-4-VEGFR-2 functional axis in D-EPCs

SDF1 α-CXCR4-VEGFR-2 is one of the most relevant axes associated with the endothelial function, and its loss leads to EPC dysfunction (EPCD) [21–25]. Hence, we investigated the expression of these mRNAs in the VN-/MCS-ND/D-EPCs and found that VN-D-EPCs effectuated significantly reduced levels *Sdf-1α*, *Cxcr-4*, and *Vegfr2* mRNAs, whereas MCS-D-EPCS showed significantly higher levels of these molecules (Fig 3G–3J). The levels of these mRNAs were similar to those in the VN-/MCS-ND-EPCs. These data suggested that the functional axis in D-EPCs is restored when cultured on MCS.

#### MCS rescues diabetes-induced EPC dysfunction by scavenging ROS and elevating NO levels in D-EPCs

Data from various cellular and functional assays demonstrated that MCS ameliorated the diabetes-induced EPCD. Hence, the next logical step would be to delve into the mechanism of rescue. Increased oxidative stress and reduced NO bio-availability are the crucial indicators of EPC dysfunction [26–29]. Therefore, we hypothesized that MCS might exhibit anti-oxidative and NO-promoting effects.

**Fig 4:**
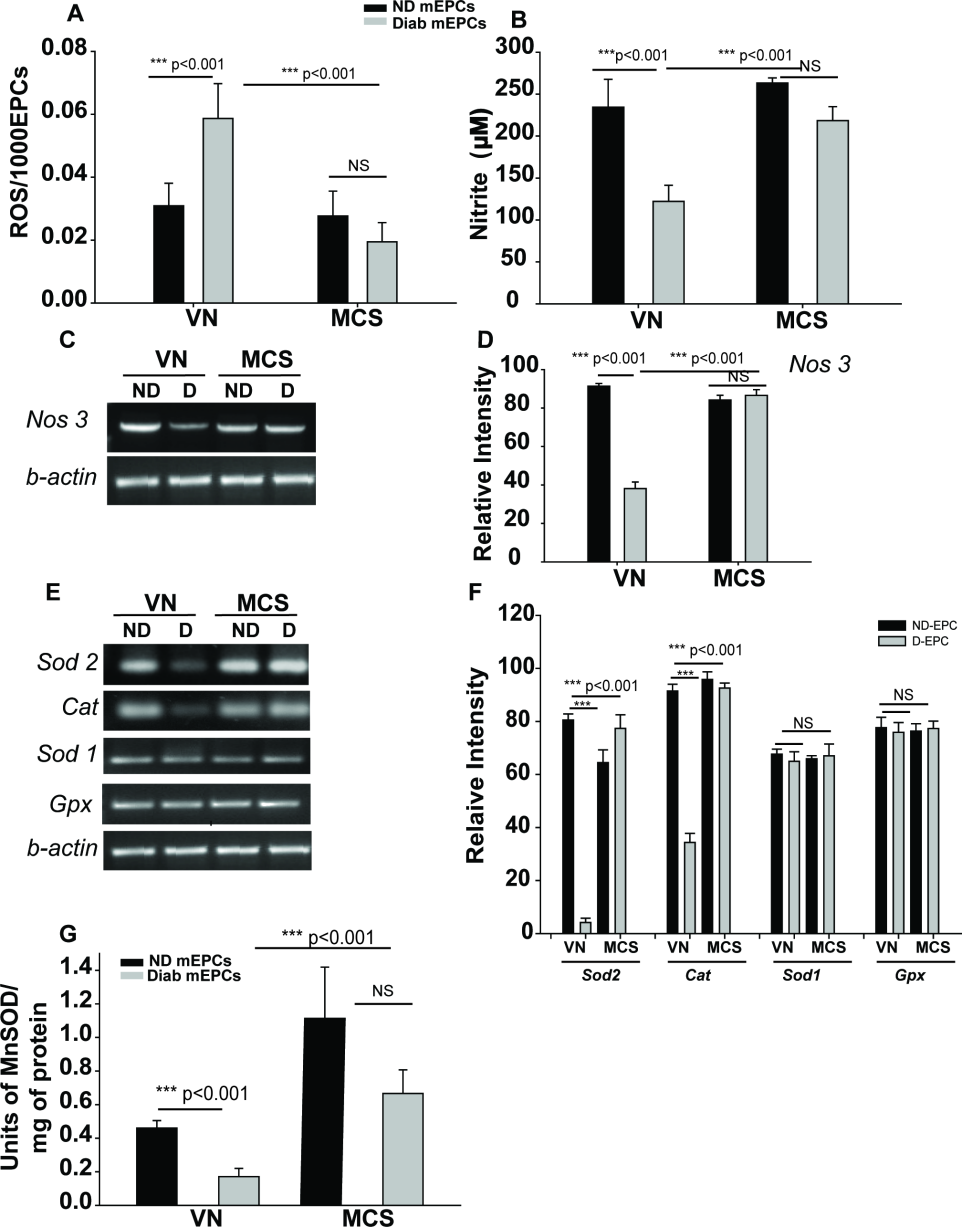
MCS rescues diabetes-induced EPC dysfunction. **(A)** ROS levels in ND- and D-EPCs cultured on VN and MCS. **(B)**Nitrite levels in the CM of ND and D-EPCs cultured on VN and MCS are represented graphically. **(C-D)***Nos3* levels in ND and D-EPCs cultured on VN and MCS. **(C)**Gel image from one representative experiment, **(D)**Mean± SD of data obtained from three independent experiments. **(E-F)** mRNA levels of *Sod2*, *Cat*, *Sod1*, *and Gpx* in ND and D-EPCs cultured on VN and MCS. The data were normalized against *β-actin*. The experiments were repeatedthree times. **(G)** MnSOD activity (units of MnSOD/mg of protein) presented in the lysate of ND and D-EPCs cultured on VN and MCS. Panel G shows densitometric analysis of *Sod2*, *Cat*, *Sod1*, and *Gpx* expression. Data are represented as a mean of triplicates from three independent experiments (n = 3) ± SD. ***P<0.001, NS (not significant).

To test this hypothesis, we estimated the cellular ROS and NO concentrations in our system. VN cultured D-EPCs showed significantly higher ROS levels as compared to VN-ND-EPCs (Fig 4A; left-hand bars), whereas MCS-D-EPCs showed a significant reduction in the ROS levels (Fig 4A; right-hand bars). The levels of ROS were similar in MCS-D- and MCS-ND-EPCs. These data revealed that MCS possesses a free radical scavenging ability, which might be attributed to the enhanced levels of free radical scavenging enzymes (Fig 4A).

Intriguingly, a significant reduction in NO levels was observed in VN-D-EPCs as compared to VN-ND-EPCs (Fig 4Bleft bars). Culturing D-EPCs on MCS restored the NO levels, indicating that MCS either prevents the depletion or stimulates the production of NO (Fig 4B; right bars). However, since the NO levels in VN and MCS cultured ND-EPCs were similar, the NO depletion might be prevented by MCS rather than the active promotion of NO production.

Since eNOS is primarily responsible for NO production in endothelial cells, we assessed eNOS-specific mRNA levels (*Nos3*) in D-EPCs. The expression of *Nos3*was significantly lower in VN-D-EPCs as compared to their ND counterparts. However, the D-EPCs cultured on MCS showed complete restoration of *Nos3*levels (Fig 4C and 4D). Thisunique feature of MCS might be largely beneficial in the rescue of EPCD for autologous EPC transplantation as a therapy in wound healing.

Since ND- and D-EPCs cultured on MCS show similar values (differences not significant, denoted as NS) in all the parameters examined, the data indicated that MCS rescues the diabetes-induced EPCD.

#### MCS rescues diabetes-induced EPC dysfunction by enhancing the levels of MnSOD and catalase

EPCs inherently possess a high resistance to ROS owing to the high levels of free radical scavenging enzymes [29]. Thus, we investigated whether MCS affects the expression of mRNAs of various antioxidant enzymes, such as MnSOD (*Sod2)*, Cu/Zn SOD *(Sod1)*, catalase (*Cat*), and glutathione peroxidase (GPx). VN-D-EPCs showed significantly low levels of *Sod2*and *Cat*; however, the levels of *Gpx* and *Sod1* were similar to those in VN-ND-EPCs (Fig 4E and 4F). Also, MCS-D-EPCs and MCS-ND-EPCs showed comparable levels of these mRNAs.

The presence or absence of mRNAs of any specific ROS scavenging enzyme is not an indication of the actual ROS scavenging potential, rather the activity of the said enzyme is the true measure. MnSOD is a known primary anti-oxidant defense mechanism in the rescue of DM-induced EPCD, and the restoration of its levels restores the functions of EPCs. Hence, we assayed the MnSOD activity in VN- and MCS-D/ND-EPCs [23, 30] and that the MnSOD activity was significantly lower in VN-D-EPCs as compared to that in VN-ND-EPCs (Fig 4G, first two bars). On the other hand, MCS-D-EPCs showed a significant restoration of MnSOD activity (Fig 4G, grey bars). These data showed that MCS boosts the expression and activity of MnSOD in D-EPCs.

Diabetes is often associated with constitutively elevated levels of ROS and pro-inflammatory cytokines. Therefore, we assessed the mRNA levels of various pro-inflammatory cytokines in the VN/MCS-ND/D-EPCs and found that VN-D-EPCs exhibited elevated levels of *Tnf-α*, *Il-1β*, and *Ifn-γ* as compared to that of VN-ND-EPCs, whereas MCS-ND- and MCS-D-EPCs showed significantly reduced levels of these mRNAs as compared to their respective counterparts (S2A and S2B Fig). These data suggested that reduction in the expression of pro-inflammatory cytokines could be one of the mechanisms underlying MCS-mediated rescue of EPCD.

EPCs actively participate in wound healing process by promoting angiogenesis and vasculogenesis [31, 32]. Hence, we examined the levels of angiogenesis-related markers in VN/MCS-ND/D-EPCs and demonstrated the enhanced levels of angiogenesis-related markers, such as Tie-1, Ang-1, PECAM, and VE-cadherin in MCS-D-EPCs as compared to their VN counterparts (S3A and S3B Fig). Interestingly, the expression of these markers was also enhanced in the MCS-ND-EPCs, suggesting that MCS actively promotes angiogenesis.

#### Topical application of MCS on diabetic wounds accelerates wound healing in diabetic mice

**Fig 5:**
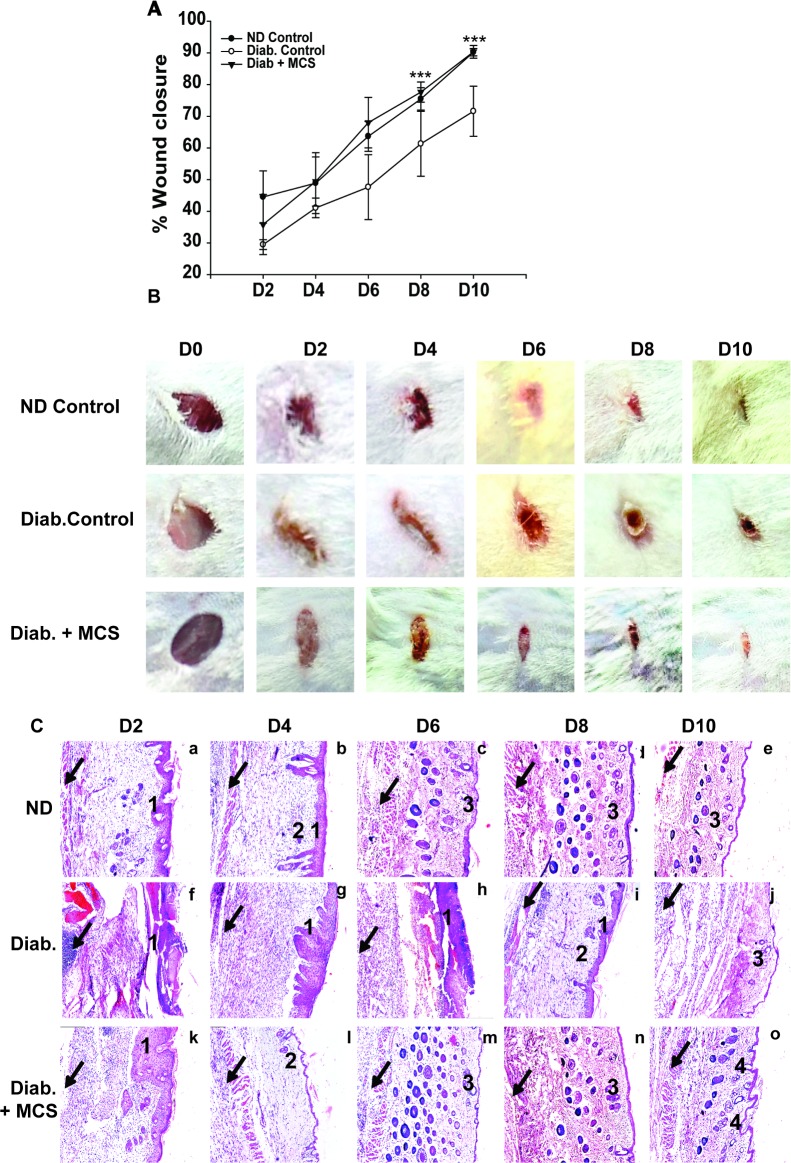
MCS accelerates wound healing process in diabetic mice. **(A)** Graphical representation of %wound closure in MCS-treated diabetic mice in comparison with ND and untreated diabetic controls. D + MCS treated wounds show significantly higher % wound closure at day10 (***P<0.001).The experiment was repeated four times (N = 4).**(B)** Imaging evidence of wound healing in mice treated with MCS from day 0–10. The images are from one representative experiment among four. At least 4 mice were analyzed for each time point. MCS-treated mice mimic normal wound healing dynamics. **(C)**Histopathological images of wound healing process. ND control [Fig 5C (images ‘a’ to ‘e’)], untreated diabetic control [Fig 5C (images ‘f’ to ‘j’), and MCS-treated diabetic mice [Fig 5C (images ‘k’ to ‘o’)] (Scale bar is 200μm). Data are representative of three independent experiments with at least 3 mice per time point. 1. Inflammatory response and macrophage infiltration. 2. Granulation. 3. Secondary follicles and epithelialization. 4. Hair follicles. Black arrows indicate panniculus carnosus.

To validate our in vitro findings in an in-vivo situation, we examined the efficacy of MCS in a diabetic wound healing model. As mentioned earlier, a standard in vivo wound healing assay was performed,and time-dependent % wound closure was determined [17]. We found that the topical application of MCS onto diabetic wounds led to an accelerated ND-like wound closure kinetics (Fig 5A). By day 10, the D-control groups showed only 67.5 ± 5.54% wound closure, which was significantly lower as compared to the ND controls (92.5 ± 1.18%), whereas MCS-treated D-wounds had achieved a near-normal closure (90.2 ± 5.74%;Fig 5B). Thus, our data demonstrated the potential of MCS in the treatment of diabetic wound healing.

#### MCS treated diabetic wounds mimic normal wound healing dynamics

Wound closure dynamics were analyzed by histopathological assessment of wound sections on days 2, 4, 6, 8, and 10 post-wounding. At day 2, all wounds showed inflammation and macrophage infiltration (marked as 1 in Fig 5C), which was maximal in control diabetic and MCS-treated diabetic wounds [Fig 5C (f and k, respectively)].By day 4, ND and D+MCS-treated wounds showed a marked reduction in inflammation and macrophage infiltration. Untreated diabetic wounds showed a reduced, but continued inflammation and macrophage infiltration up to day 8. The initiation of granulation (marked as 2 in Fig 5C) was seen in ND and MCS-treated D wounds [Fig 5C (b and i, respectively)]. Between days 6 and8, secondary structure formation and epithelialization was (marked as 3 in Fig 5C) evident in ND [Fig 5C(c, d)] and MCS-treated D wounds [Fig 5C (m, n)]. The untreated D wounds showed epithelialization, but no secondary structure formation [Fig 5C (h, i)]. By day 10, the untreated D wounds showed poor and delayed wound healing without any hair follicle formation [Fig 5C(j)]. ND wounds showed initiation of hair follicle formation by day 6, and interestingly, the MCS-treated D wounds showed a remarkably increased hair follicle formation (marked as 4 in Fig 5C-m). Overall, the data showed that MCS-treated D wounds followed normal wound healing kinetics [Fig 5C (o and e, respectively)].These data clearly demonstrated the efficacy of MCS in diabetic wound healing in vivo.

#### Mechanism of MCS-mediated rescues of EPCD

**Fig 6:**
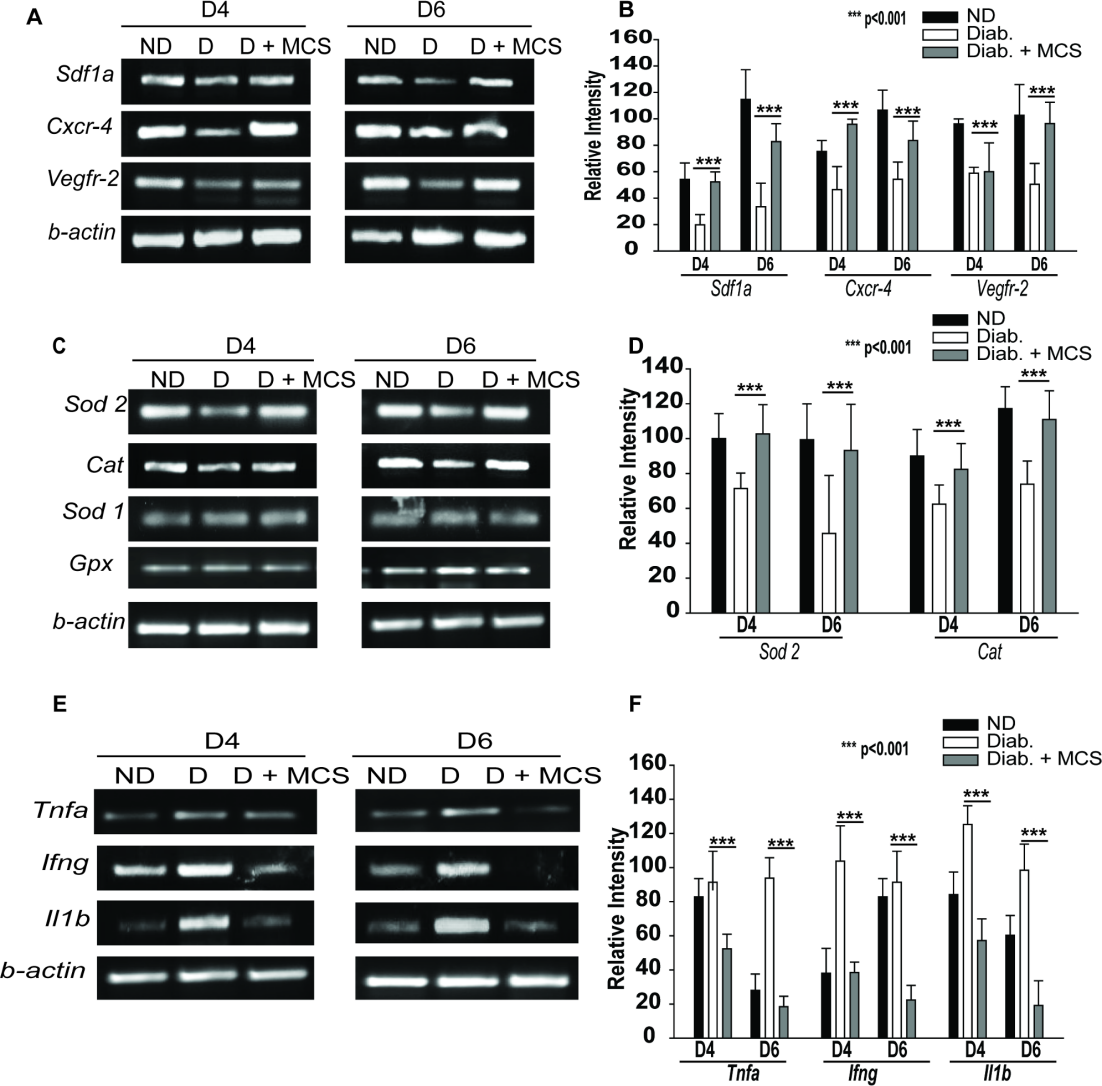
Mechanism of MCS-mediated EPC rescue. **(A-B)** cDNAs prepared from wound biopsies of ND, D, and D+ MCS mice were subjected to RT-PCR analysis. Expression of *Sdf-1a*, *Cxcr4*, *and Vegfr2* in ND, D, and D + MCS-treated diabetic wounds is shown. **(A)** Gel image of one representative experiment, **(B)**Densitometric analysis of data obtained in three independent experiments. **(C-D)** Levels of *Sod2*, *Cat*, *Sod1*, and *Gpx* in the wounds. **(C)**Gel image from one representative experiment, **(D)** Densitometric analysis data obtained from three independent experiments. **(E-F)** MCS downregulates the expression of *Tnf-α*, *IL-1β*, and *Ifn-γ* in diabetic wounds. **(E)**Gel image from a representative experiment, **(F)**Densitometric analysis data obtained from three independent experiments. Data in all experiments were normalized to that of *β-actin*. ***P<0.001.

To elucidate the mechanism by which MCS accelerates diabetic wound healing process, we examined the effect of local application of MCS on *Sdf-α*, *Cxcr-4*, and *Vegfr-2*functionality axes by RT-PCR analyses of wound biopsies. The MCS treatment led to an enhanced expression of *Sdf1a*, *Cxcr4*, and *Vegfr2* mRNAs in the wound tissue (Fig 6A and 6B). Consistent with the in vitro data, our in vivo data also showed elevated levels of *Sod2*and *Cat* mRNA levels and reduced levels of mRNAs of inflammatory cytokines in the wound biopsies (Fig 6C and 6D). However, the levels of *Sod1* and *Gpx* were unaffected. Also, the levels of mRNAs for various inflammatory cytokines, such as *Tnf-α*, *Ifn-γ*, and *Il-1β* were reduced in the MCS-treated wounds (Fig 6E and 6F). These data showed that MCS boosts wound healing by rescuing the diabetes-induced EPCD.

**Fig 7:**
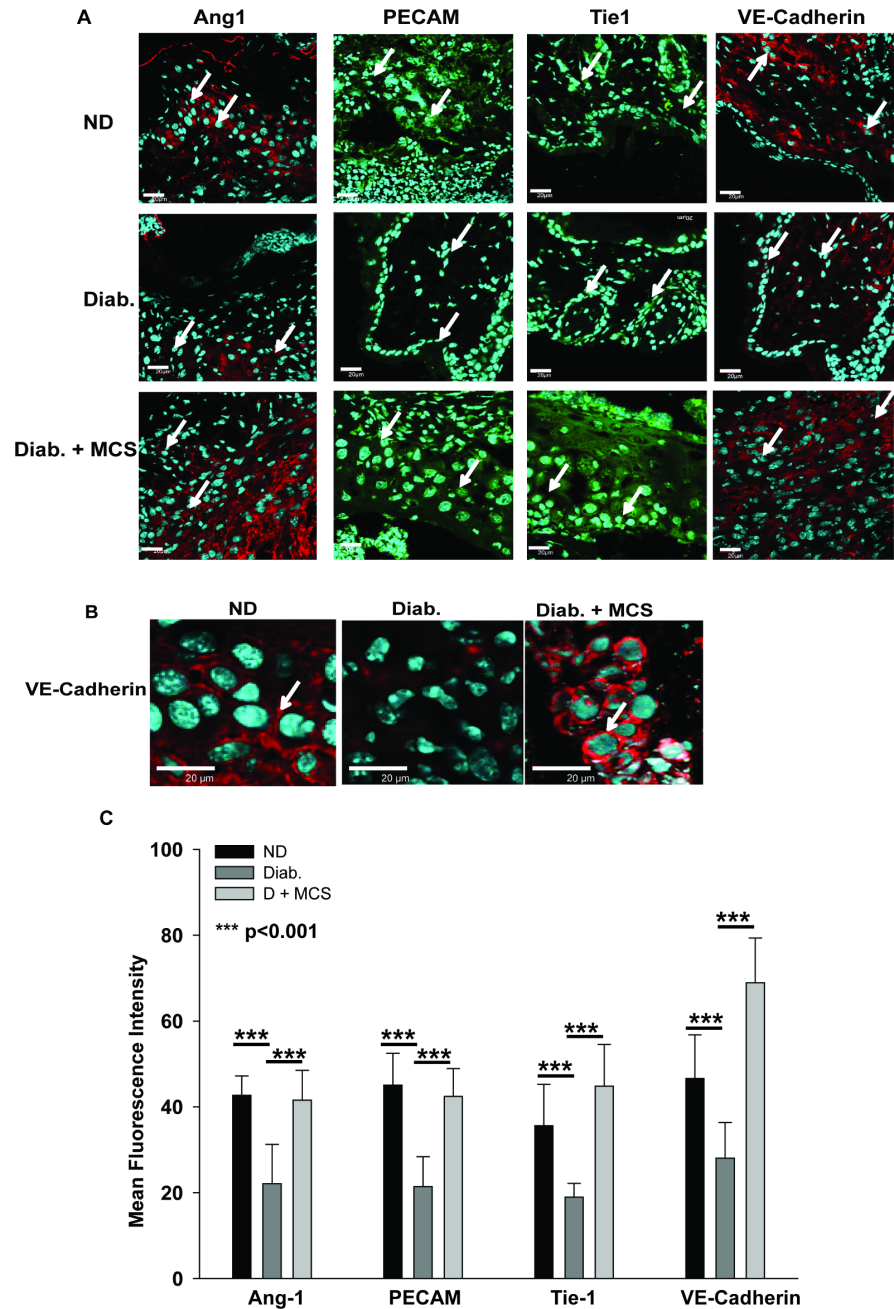
MCS stimulates angiogenesis. **(A)** Cryo-sections of ND, D, and D + MCS wounds were stained for Ang-1 (Cy3), PECAM (FITC), Tie1 (FITC), and VE-cadherin (Cy3) and counterstained with DAPI to demarcate the nuclei. Scale bars for images = 20 μm; the data are representative of 4 mice in each group. White arrows indicate the areas of positive signals for Ang-1, PECAM, Tie1, and VE-cadherin expression. **(B)**Distinct VE-cadherin-positive cell junctions within the wound sections. White arrows indicate cell-cell junctions.**(C)** Mean fluorescence intensity data of images shown in **(A)** obtained from four independent experiments. ***P<0.001.

Since angiogenesis is one of the key steps in the wound healing cascade, we measured *Ang1/2* and *Tie1/2* mRNA levels in the wound sections and found that diabetes-induced reduction in *Ang1/2* and *Tie1/2*mRNA expression was rescued by MCS treatment (S4 Fig). In addition to these effects, MCS treatment also elevated the expression of Tie1, PECAM, and V-cadherin in the wounds (Fig **7**A, **7**B and **7**C). These data suggested that MCS accelerates diabetic wound healing by boosting angiogenesis.

#### Identification of Klotho as one of the active constituents of MCS

Klotho is a known anti-aging hormone. The overexpression of Klotho leads to increased *SOD2* (MnSOD) expression imparting resistance to increased oxidative stress in the kidney of STZ-induced diabetic rats [33–35]. It also protects vascular endothelial cells from H_2_O_2_-induced apoptosis and senescence and is involved in angiogenesis and vasculogenesis [36]. Our LCMS data, albeit qualitative, yielded Klotho as one of the most consistent hits (S1 Table), and therefore, we studied its expression in the VN-/MCS ND/D EPCs by immunofluorescence staining. VN-ND-EPCs displayed a significantly higher expression of Klotho as compared to VN-D-EPCs at both protein (S5A and S5B Fig) and mRNA levels (S5C and S5D Fig). Importantly, MCS-D-EPCs showed that the expression of Klotho was similar to that of MCS-D-EPCs.

These data indicate that Klotho might be one of the active constituents of MCS involved in the rescue of DM-EPCD. Consecutively, MCS-D-EPCs showed increased *Sod-2* expression (Fig 4E and 4F), indicating that the presence of Klotho in MCS induces *Sod-2* expression in D-EPCs leading to resistance to diabetes-induced oxidative damage.

In summary, the current data showed that MCS rescues DM-EPCD by reducing the levels of ROS, increasing the levels of NO, reducing the inflammation, enhancing the angiogenic properties, and restoring the expression of the CXCR4-SDF1α-VEGFR2 axis in D-EPCs. These data illustrate the potential application of MCS in the treatment of diabetic wounds and non-healing ulcers.

## Discussion

Cellular secretomes play a key role in regenerative therapy in various disease conditions such as cardiovascular disorders, ischemic heart disease, and diabetic wound healing [37]. Current procedures used for harvesting the secretome typically involve the collection and enrichment of the components secreted in the CM of cell cultures, which often leads to the loss of labile active components [38]. Although hydrogels are suitable for accelerated wound healing due to their analgesic and anti-inflammation properties [39], harvesting them in a hydrogel consistency from CM is ratherchallenging as most of them are highly water-soluble. To overcome these drawbacks, we used PCG-nanofiber matrix for culturing the bone marrow-derived mononuclear cells that offer a 3D meshwork for complex cellular interactions ensuing formation and entrapment of secretomes (MCS). Herein, we showed that MCS couldbe used as a biomaterial for the optimal growth of EPCs, even from a diabetic source. The use of PCG for harvesting another biomaterial MCS is rather a novel approach. The benefits offered by the system described in the present study include prevention of washing away of the secreted products and ease of harvesting of the secretome in a hydrogel-like consistency without compromising the concentration and activity of the active components. Interestingly, the supernatant collected from these cultures did not show wound healing activity similar to that of MCs, thereby indicating entrapment of all active components in the matrix.

The current immunohistochemistry data showed that MCS formation could be primarily attributed to the interactions of EPCs, MSCs, and pericytes, through contribution from the unidentified minor fraction cannot be negated at present. However, such secretome was not formed when purified individual cell types were cultured on PCG matrix, or a mixed population was generated without the PCG matrix or on PLLA matrix, suggesting that the heterogeneity of both culture and matrix composition is crucial for MCS formation (under IPR).

For the structural characterization of MCS, we used cryo-SEM as it is a technique used for the ultra-structure of liquids, semi-liquids, or gels in their native, chemically unaltered and hydrated state. The structural characterization of MCS by cryo-SEM revealed a 3D bone marrow-like architecture, which might be beneficial as a substrate for culturing different cell types, including stem cells. Thus, we proposed an additional investigation of this aspect in future studies.

Diabetes-induced hyperglycemia affects the overall yield of EPCs. Hence, we evaluated the efficacy of MCS in culturing diabetic EPCs, which are otherwise difficult to culture due to compromised viability, adhesion, proliferation, and colony formation potential. As evident from our data, MCS form a better substrate for culturing D-EPCs as compared to other commonly used substrates, such as like VN, FN, and collagen. Although matrigel was found to similar to MCS, it is prepared from neoplastic cells, whereas MCS is a product of normal cells, and hence, would be applicable in clinical settings.

D-EPCs fail to mobilize and migrate from bone marrow towards the wound areas due to defective chemotactic migration. Hence, we tested the functionality of D-EPCs cultured on MCS and found that both impaired tube formation and migratory potential of D-EPCs were reversed when cultured on MCS, emphasizing the role of MCS in the rescue of diabetes-induced EPCD.

Hyperglycemia-induced oxidative stress and reduced availability of nitric oxide (NO) are the primary factors responsible for EPCD [27, 40, 41, 42]. The present study showed that MCS rescues the ROS levels in D-EPCs and also prevents NO depletion. Furthermore, MCS reduced the level of cellular ROS by enhancing the levels of *SOD2* and *Cat*. The MnSOD activity was elevated in MCS-D-EPCs. Also, MCS restored the levels of *Sdf1a*, *Cxcr4*, and *Nos3*in D-EPCs. MCS-EPCs (ND/D) showed depleted levels of mRNAs specific for the three inflammatory cytokines (*TNF-α*, *Il-1β*, *IFN-γ*), indicating the robust anti-inflammatory activity. However, this aspect necessitates further investigation in order to shed light on how the MCS achieves this effect. We found that the expression of *Nos3* (eNOS) was reduced that of the control VN-D-EPCs, indicating that the expression was adversely affected in a diabetic scenario. Interestingly, D-EPCs cultured on MCS showed restored *Nos3* levels. Taken together, these unique properties of MCS and our data showed that MCS was beneficial in the rescue of EPCD for autologous transplantation.

Importantly, the in vivo diabetic wound healing data demonstrated the efficacy of MCS. A topical application of MCS accelerated the scar-free healing of diabetic wounds. A majority of the cell therapies either employ direct transplantation of normal EPCs collected from allogeneic sources or in vitro cultured D-EPCs for autologous transplantation. However, the major limitation of these cell therapies is the need of culturing cells as per requirement. Also, the cryopreservation of fastidious cells, such as EPCs is challenging. Thus, the loss of cell viability and number during transplantation, the need of in vitro culture and rescue of EPCs before use, and immunological incompatibility issues renders the therapy tedious and less cost-effective. Since MCS is a storable, cell-free, and ready-to-use biomaterial, its application is not only time- and cost-efficient but also immunologically safe.

Another interesting observation was the increased formation of hair follicles in the diabetic wounds treated with MCS in this study. Usually, the healing wounds do not show hair follicles up to day 14[36]. However, the analysis of multiple sections showed that in ND mice, the hair follicle growth was evident by day 6 post-wounding. This phenomenon might be related to the difference among the mouse strains. The diabetic wounds failed to show hair follicle formation up to day 10. Intriguingly, the diabetic wounds treated with MCS showed the formation of a large number of hair follicles by day 6. Whether MCS stimulatesthe hair root stem cells needs to be examined further.

Klotho is a maintenance factor for endothelium-derived NO, EPC differentiation and mobilization, and neo-vascularization [43]. Under hyperglycemic conditions, reduced levels of Klotho and phosphorylated forkhead box O proteins (FOXOs)lead to impaired angiogenesis, NO formation, and EPC migration [44,45]. We observed reduced levels of Klotho in VN-D-EPCs, whereas MCS could restore the levels at par with that of ND-EPCs. In the proteomic analysis of MCS, Klotho was found to be a consistent hit, suggesting that it was provided by MCS and might be involved in increased resistance to oxidative stress, enhanced *SOD2* levels, increased angiogenesis in MCS-treated diabetic mice alone, or triggering other downstream pathways that eventually lead to the rescue of EPCD.

A delayed wound healing and impaired granulation formation have been observed in Klotho-deficient mice [46]. Wounds in these mice also showed a high expression of inflammatory cytokine related mRNAs. Consistent with these reports, the application of MCS also resulted in accelerated wound healing and reduction of mRNAs related to inflammatory cytokines. However, whether Klotho alone shows such activity or requires the cooperative action of other components present in the MCS is yet to be elucidated. The skin of Klotho-deficient mice shows signs of aging [47]. The current data in conjunction with this report suggested that in addition to the application in diabetic wound healing, MCS might also be valuable in anti-aging treatments of the skin. However, this aspect needs further exploration.

## Conclusion

MCS, a biomaterial-derived biomaterial, is a ready-to-use cell-free material that can rescue the diabetes-induced EPCD. It can be applied as a salve to diabetic wounds to facilitate healing in vivo.

## Supporting information

S1 Fig **(A)** BM-MNCs were cultured on PCG-nanofiber matrix for 14 days. Subsequently, MCS formation cells were stained for Ac-LDL uptake (Alexa flour 488) (EPC marker), CD 146 (Cy3) (MSC marker), and CD 31 (FITC), and Ang-1 (CY3) (Pericytes marker). The nuclei are stained with DAPI. Each cell type was counted manually in at least 10non-overlapping fields, and the percentage of each contributing cell type was calculated. CM collected during MCS formation was tested for the ability to support ND-and D-EPCs growth by cellular assays. 14 D-EPCs were cultured on VN, MCS, and MCS-CM and assessed for % viability **(B),** cellular adhesion **(C),**and proliferation potential **(D),** and colony formation **(E).** CM does not support ND- or D-EPC growth. Data are represented as mean of three independent experiments (N = 3) ±SD. ***P<0.001.
(TIF)

S2 Fig **(A)** VN/MCS-D-EPCs were subjected to RT-PCR analysis to evaluate the levels of *Tnf-α*, *Il-1β*, and *Ifn-γ*. **(B)**Densitometric analysis of data obtained from three independent experiments. MCS-D-EPCs showed significantly reduced levels of mRNAs of these cytokines as compared to their control counterparts. ***P<0.001.
(TIF)

S3 Fig **(A)** MCS-D-EPCs show enhanced expression of angiogenic markers, such as Tie 1 (Cy3), Ang-1 (Cy3), PECAM (FITC), and VE-Cadherin (Cy3) as compared to VN-D-EPCs. **(B)** Mean fluorescence intensity of the data in panel A. Images from three independent experiments were analyzed. Nuclei were stained with DAPI. Scale bar = 20μm. ***P<0.001, NS: not significant.
(TIF)

S4 Fig **(A)** Biopsies collected from ND, D, and D+MCS wounds were subjected to RT-PCR analyses to examine the levels of *ang1/2* and *TIE1/2*. Panel B shows densitometric analysis obtained from three independent experiments. Data show that the topical application of MCS on diabetic wounds enhances the expression of these mRNAs. *P<0.05, ***P<0.001.
(TIF)

S5 Fig **(A)** Confocal microscopy analysis of EPCs immuno-stained with antibodies to Klotho. Right-hand panel depicts mean fluoresce intensity. **(B)** Expression of Klotho-specific mRNA (*Kl*) in the EPCs. The right-hand panel depicts the densitometric analysis of the bands. Data are represented as mean ± SD of three independent experiments. ***P<0.001.
(TIF)

S1 Table Chemical characterization of MCS by LCMS. (XLSX)
