# Supplementary figures and images for: Matrix-entrapped cellular secretome rescues diabetes-induced EPC dysfunction and accelerates wound healing in diabetic mice
Source: PLoS One. 2018 Aug 28;13(8):e0202510. doi: 10.1371/journal.pone.0202510 (PMC6112628; doi:10.1371/journal.pone.0202510)

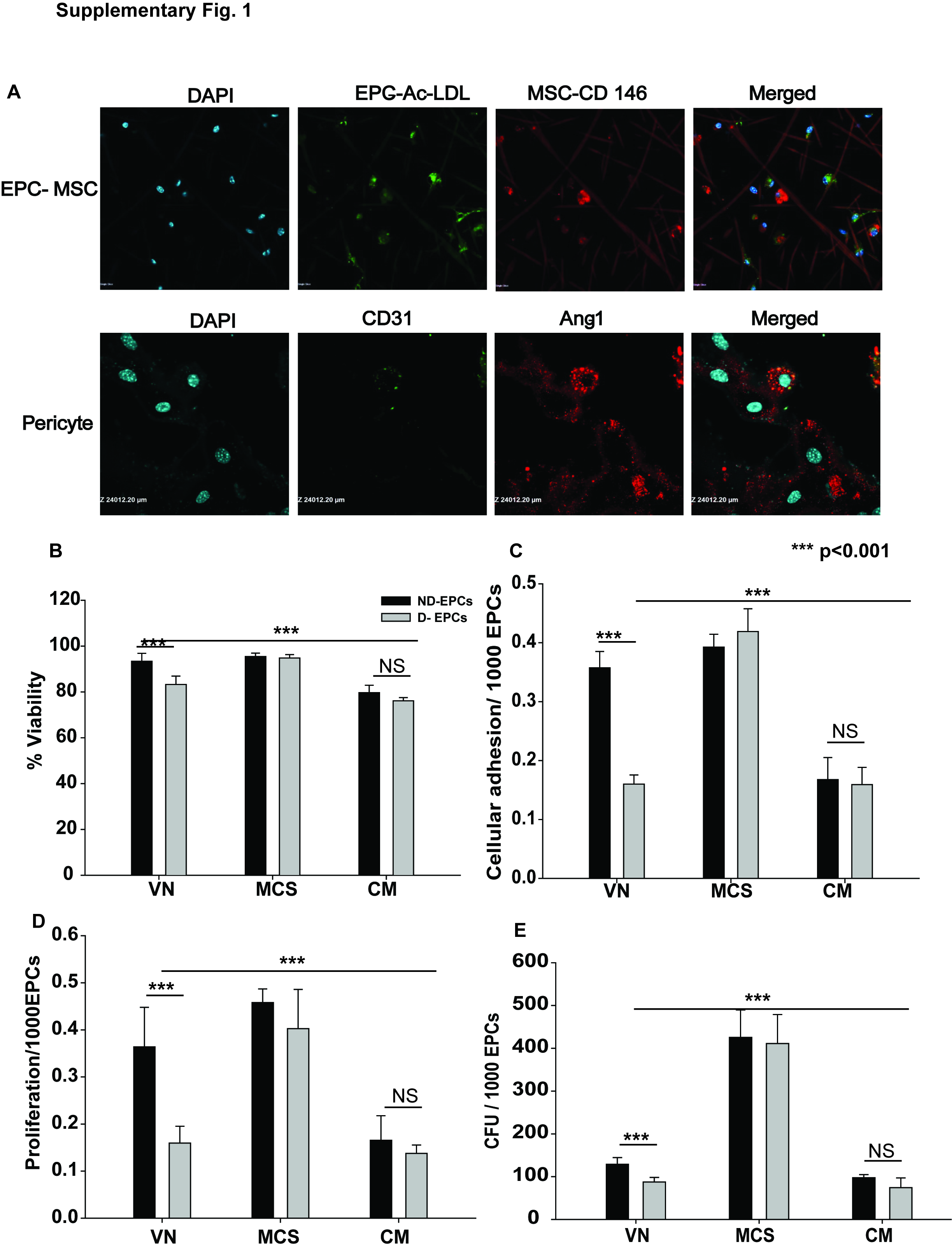

Supplement: S1 Fig — (A) BM-MNCs were cultured on PCG-nanofiber matrix for 14 days. Subsequently, MCS formation cells were stained for Ac-LDL uptake (Alexa flour 488) (EPC marker), CD 146 (Cy3) (MSC marker), and CD 31 (FITC), and Ang-1 (CY3) (Pericytes marker). The nuclei are stained with DAPI. Each cell type was counted manually in at least 10non-overlapping fields, and the percentage of each contributing cell type was calculated. CM collected during MCS formation was tested for the ability to support ND-and D-EPCs growth by cellular assays. 14 D-EPCs were cultured on VN, MCS, and MCS-CM and assessed for % viability (B), cellular adhesion (C),and proliferation potential (D), and colony formation (E). CM does not support ND- or D-EPC growth. Data are represented as mean of three independent experiments (N = 3) ±SD. ***P<0.001. (TIF) [file pone.0202510.s001.tif]

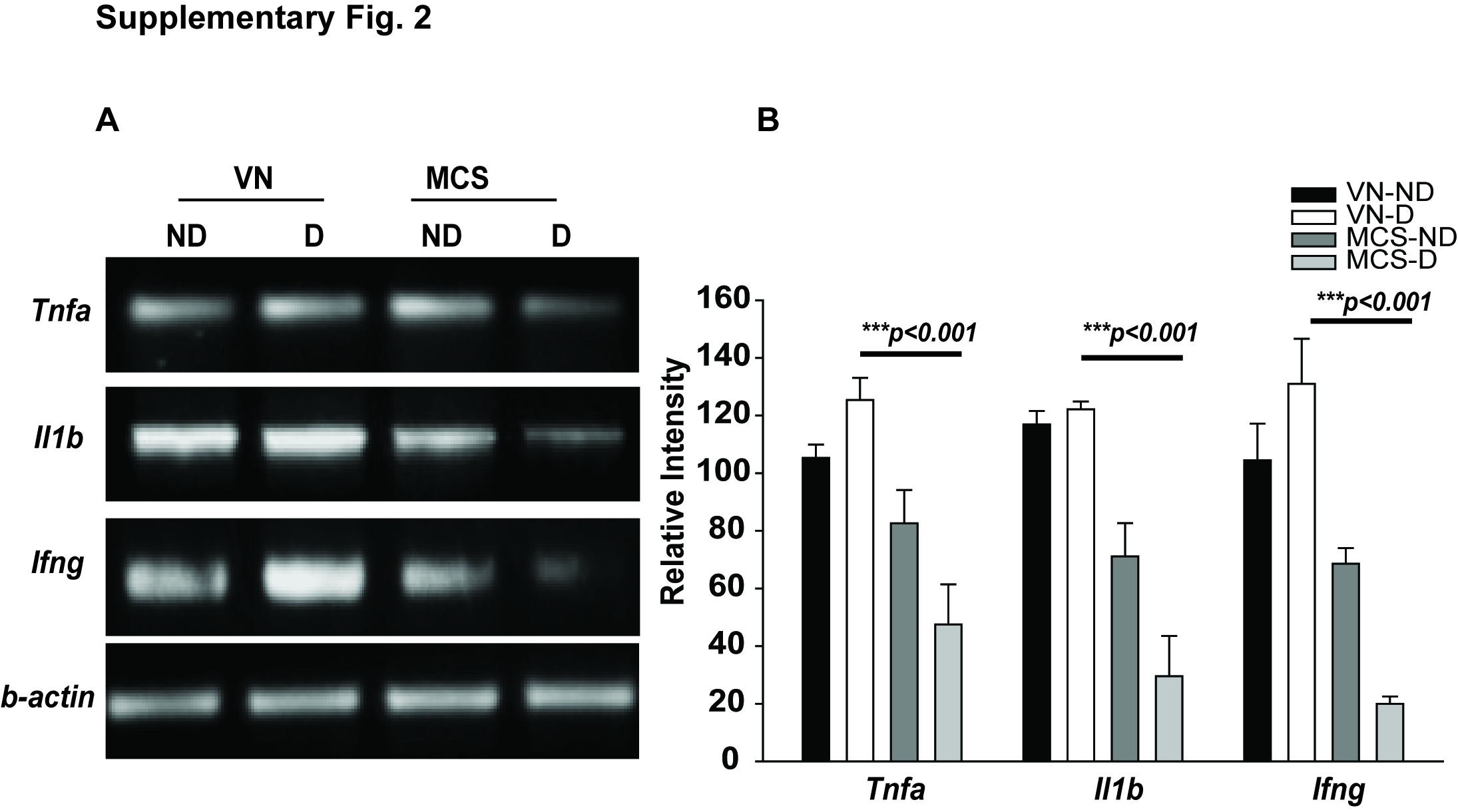

Supplement: S2 Fig — (A) VN/MCS-D-EPCs were subjected to RT-PCR analysis to evaluate the levels of Tnf-α, Il-1β, and Ifn-γ. (B)Densitometric analysis of data obtained from three independent experiments. MCS-D-EPCs showed significantly reduced levels of mRNAs of these cytokines as compared to their control counterparts. ***P<0.001. (TIF) [file pone.0202510.s002.tif]

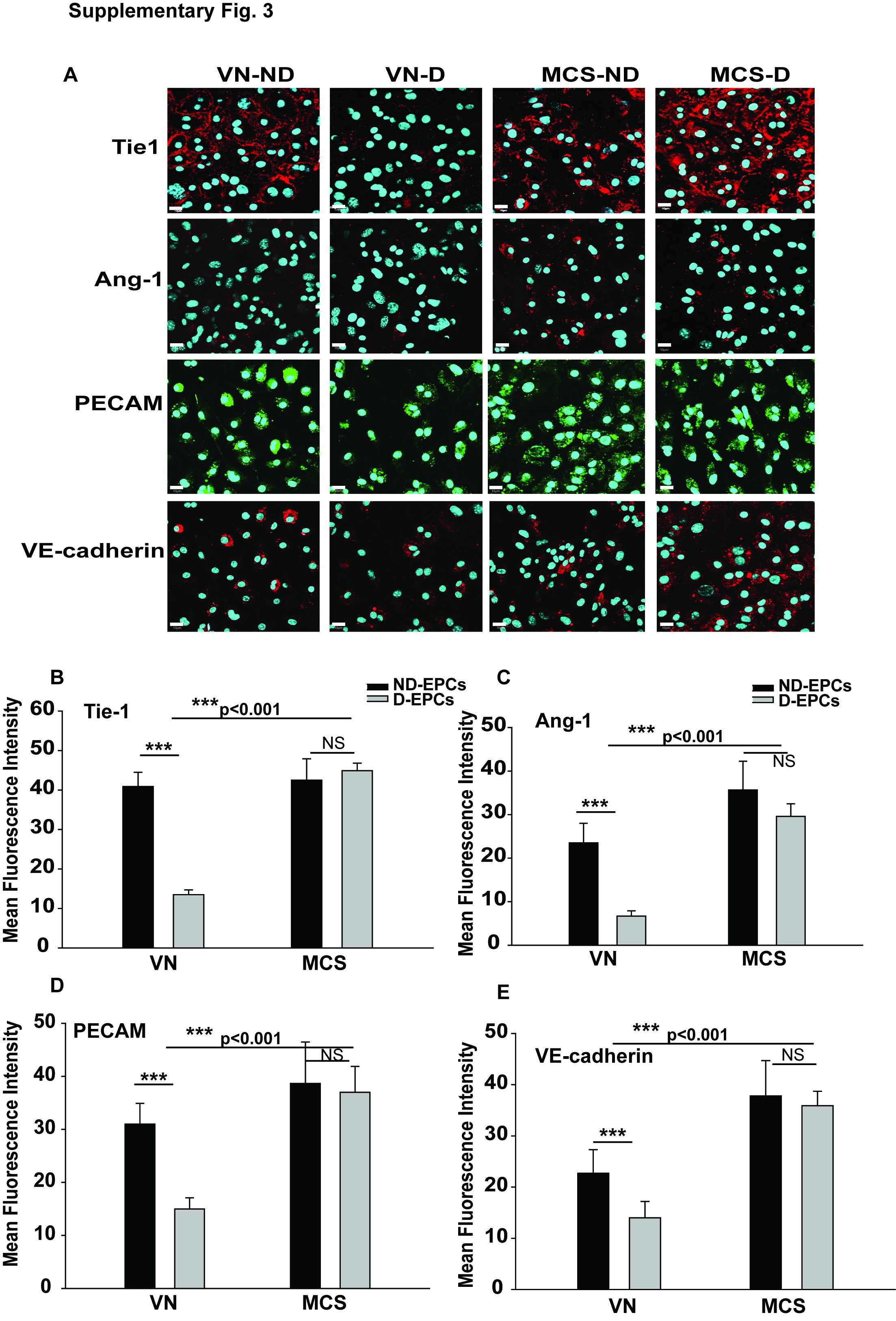

Supplement: S3 Fig — (A) MCS-D-EPCs show enhanced expression of angiogenic markers, such as Tie 1 (Cy3), Ang-1 (Cy3), PECAM (FITC), and VE-Cadherin (Cy3) as compared to VN-D-EPCs. (B) Mean fluorescence intensity of the data in panel A. Images from three independent experiments were analyzed. Nuclei were stained with DAPI. Scale bar = 20μm. ***P<0.001, NS: not significant. (TIF) [file pone.0202510.s003.tif]

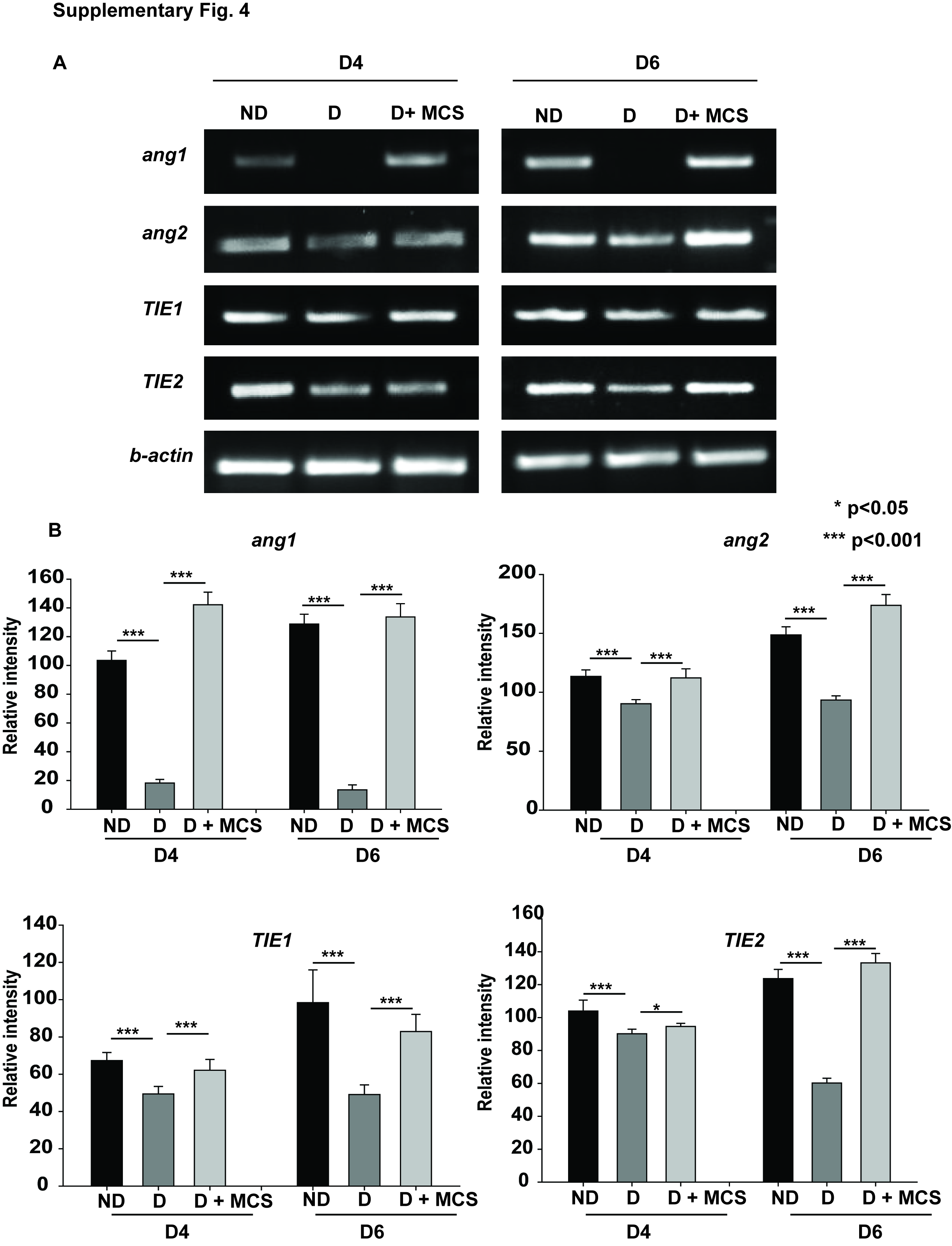

Supplement: S4 Fig — (A) Biopsies collected from ND, D, and D+MCS wounds were subjected to RT-PCR analyses to examine the levels of ang1/2 and TIE1/2. Panel B shows densitometric analysis obtained from three independent experiments. Data show that the topical application of MCS on diabetic wounds enhances the expression of these mRNAs. *P<0.05, ***P<0.001. (TIF) [file pone.0202510.s004.tif]

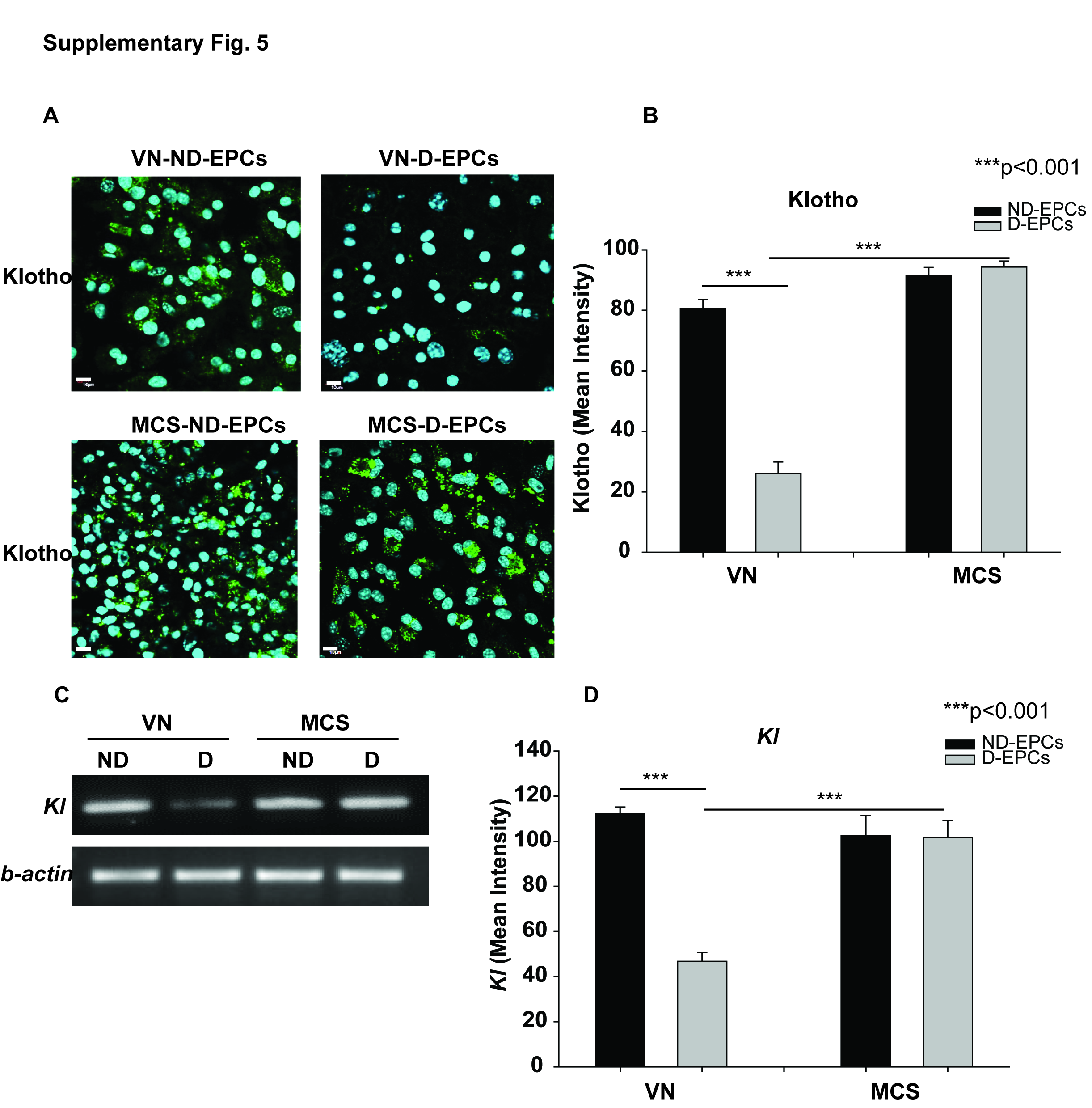

Supplement: S5 Fig — (A) Confocal microscopy analysis of EPCs immuno-stained with antibodies to Klotho. Right-hand panel depicts mean fluoresce intensity. (B) Expression of Klotho-specific mRNA (Kl) in the EPCs. The right-hand panel depicts the densitometric analysis of the bands. Data are represented as mean ± SD of three independent experiments. ***P<0.001. (TIF) [file pone.0202510.s005.tif]
